# Successful Separation of Omphalopagus Twins: A Case Report

**Published:** 2016-01-01

**Authors:** Prashant S Patil, Paras Kothari, Abhaya Gupta, Geeta Kekre, KV Dikshit, Ravi Kamble, Shahaji Deshmukh

**Affiliations:** L.T.M.G. Hospital, Sion, Mumbai, Maharashtra, India

**Keywords:** Conjoined twins, Omphalopagus

## Abstract

Omphalopagus twins are conjoined twins sharing part of gastrointestinal system and abdominal wall. These types of twins have best chances of survival if successfully separated. We report a case of successfully separated omphalopagus twins at day six of life.

## CASE REPORT

A 24-year-old multi-para underwent elective caesarian section in our hospital and delivered female omphalopagus twins (Fig.1). The combined birth weight of babies was 4.9 kg. The babies were designated as "twin A" and "twin B". The babies were fused from epigastrium to umbilicus and had a single umbilical cord. The bridge of tissue was firm, 2 cm thick and 5 cm in length. Both babies cried immediately after birth and passed urine and meconium. 

**Figure:**
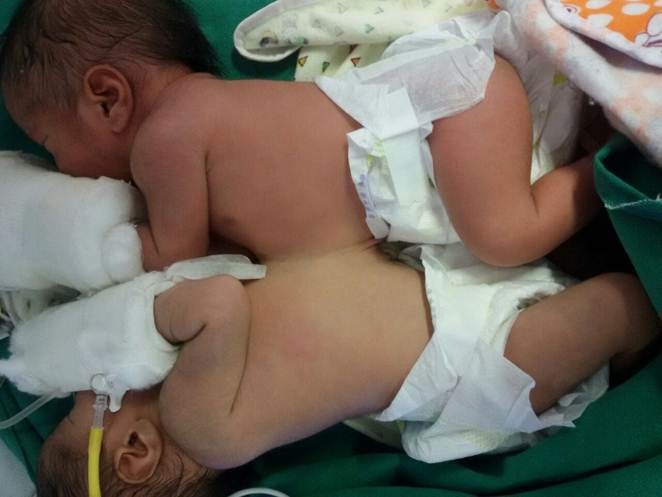
Figure 1: Omphalopagus twins at birth

Ultrasonography of abdomen revealed fusion of liver and separate organ systems. Doppler ultrasound confirmed that the hepatic veins, portal vein and inferior vena cava of both twins were separate. Contrast CT scan showed differential enhancement of livers of both twins (Fig. 2). The extra hepatic biliary system of both twins was separate with two gall bladders and common bile ducts. Rest of the organs were not shared. A 2-D echo study of twin A revealed 10 mm muscular VSD while twin B had small ASD with PDA. Blood investigations revealed Hb of 12.3 gm% for Twin A and 14.2 gm% for Twin B, with normal biochemistry values for both.

**Figure:**
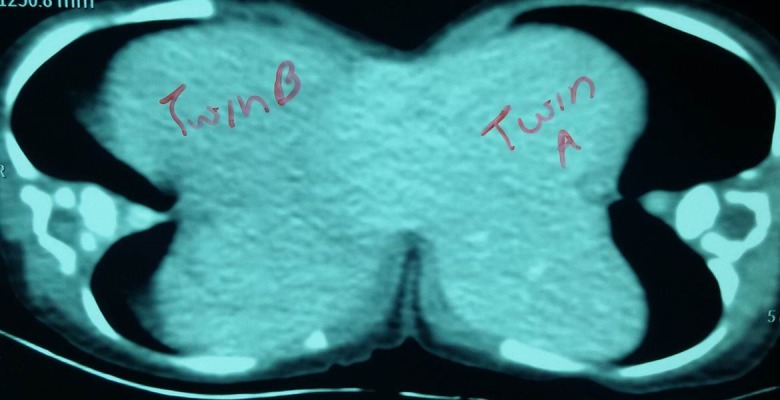
Figure 2: Contrast CT-scan showing differential enhancement of livers of both twins.

Written informed consent was obtained from parents with the provision of saving the baby with better chance of survival if situation arises. The surgical plan, the anesthesia plan, location of anesthesia and surgical equipment, patient positioning and plan for repositioning after physical separation were discussed. Blood and blood products including fresh frozen plasma, platelets were reserved for surgery. Babies were taken for surgery on single operation table keeping one more table ready for closure of abdomen after separation. Separate anesthesia and surgery teams kept ready for each baby. Baby A was induced first followed by baby B. After anesthetic preparation was complete, the twins were lifted and surgically prepped on the posterior side first, placed on sterile sheets and then prepped anteriorly. Incision was taken on the connecting bridge of tissue. Skin and muscles were cut. Connecting part of liver was carefully divided using harmonic scalpel (Fig. 3). Vital parameters of twin B who had minor cardiac anomaly showed slight deviation from normal but ultimately came back to normal by end of surgery. About 25 cc of packed red blood cells was transfused to each twin intraoperatively. After two and half hours of surgery, the twins were finally separated and twin B was taken to another table. Cut edges of liver of each baby were approximated with Vicryl 2-0. Abdominal drain placed in each baby and abdominal wall was closed. Both babies tolerated procedure well and extubated on table and shifted to pediatric surgery intensive care unit. Intravenous antibiotics were given till tenth post-op day. Oral feeds were started on post-op day four; abdominal drain was removed on post-op day five. Baby B had wound infection which was managed conservatively. Both babies were discharged on post-op day 12. Vaccination was done during hospital stay. Both babies have gained weight and on follow-up for five months.

**Figure:**
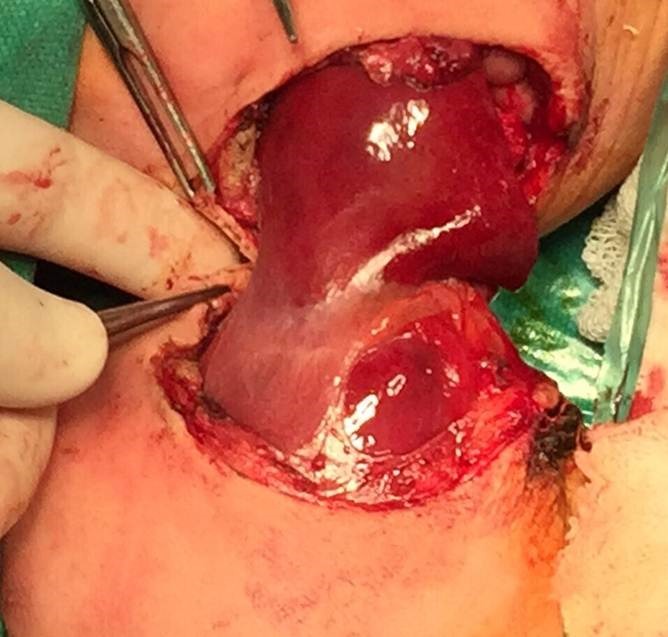
Figure 3: Connecting part of liver between twins

## DISCUSSION

Conjoined twins prevail from 1:50,000 to 1:200,000; around 250 surgical separations have been reported so far. The largest series reported includes 17 sets of twins by Spitz et al. [1] and 25 sets by Cywes et al. [2]. The separation of conjoined twins presents a unique challenge to pediatric surgeons because of its rarity. Although omphalopagus twins have the best chances of survival, adequate team management and preoperative planning is required. Radiological investigations are needed for the evaluation of the shared organs, presence of anomalies, presence and extent of cross circulation.[3] The timing of surgery is controversial; however, a delay of few months gives better chances of survival. [1] Early separation is indicated when one twin threatens the life of the other. [1,4] Although major cardiac anomalies are contraindications of separations, variations in the cardiac functions have been observed with otherwise normal hearts as in our case.

Conjoined biliary tract is reported in 25% of cases of omphalopagus twins. [5] The routine evaluation of cross circulation is performed using many methods like Tc-99m microcolloidal human serum albumin (HSA),Tc-99m HIDA, [6] injection of indigo carmine and the examination of its excretion in urine of the other twin. One method of evaluating cross circulation is contrast CT scan, [1] which in our case showed a watershed zone in the left side of liver bridge. Some have advocated glucose tolerance test for the evaluation of parasitism. [7] Intravenous fluorescein can be used to demarcate the large liver juncture. [7]

Surgical separation should take the following into account: shared organs, soft tissue and bony defects that ensue after separation, age of the child and associated anomalies. Accurate preoperative investigations, a team approach, previous experience and meticulous operative and postoperative management contribute to the success rate. [6] After separation, there are often large areas devoid of skin; despite the use of preoperative tissue expansion, primary wound closure is not always possible, increasing the risk of postoperative sepsis in these individuals. [8] We could close the abdominal wound primarily in both babies though twin B had wound infection which required daily dressing. The long-term survival and outcome in relation to education and psychological outcome in separated twins is good. [9]

## Footnotes

**Source of Support:** Nil

**Conflict of Interest:** Nil

## References

[1] Kingston CA, McHugh K, Kumaradevan J, Kiely EM, Spitz L. Imaging in the preoperative assessment of conjoined twins. Radiographics. 2001; 21:1187-2082. 10.1148/radiographics.21.5.g01se011187 11553825

[2] Cywes S, Millar AJ, Rode H, Brown RA. Conjoined twins-the Cape Town experience. Pediatr Surg Int. 1997; 12:234-48. 10.1007/BF01372142 9099638

[3] Spitz L, Kiely EM. Experience in the management of conjoined twins. Br J Surg. 2002; 89:1188-92. 10.1046/j.1365-2168.2002.02193.x 12190687

[4] Lai HS, Lee PH, Chu SH, Chen MT, Lin TW, Duh YC, et al. Successful early separation of a premature xipho-omphalopagus conjoined twins: A case report. Can J Surg. 1997; 40:141-2. PMC3952978 9126129

[5] Ray AK, Mukherjee NN, Mukherjee G, Patra R, Ghosh DK. Separation of thoraco-omphalopagus siamese twin. Indian J Pediatr. 2004; 71:755-7. 10.1007/BF02730669 15345880

[6] Rubini G, Paradies G, Leggio A, D'Addabbo A. Scintigraphy in assessment of the feasibility of separation of a set of xipho-omphalopagous conjoined twins. Clin Nucl Med. 1995; 20:1074-8. 10.1097/00003072-199512000-00008 8674294

[7] Kenigsberg K, Harper RG. Separation of omphalopagus twins. J Pediatr Surg. 1982; 17:255-8. 10.1016/s0022-3468(82)80007-9 6213752

[8] Aird I. Conjoined twins: Further observations. Br Med J. 1959; 1:1313-5. 10.1136/bmj.1.5133.1313 PMC1993488 13651722

[9] El-Gohary MA, Mohyuddin Z, Lakshmi B, Sedaghatian R. Separation of xipho-omphalopagus twins. Pediatr Surg Int. 1988; 3:76-8.

